# Supplementary material for: Occurrence and Dietary Exposure Assessment to Enniatin B through Consumption of Cereal-Based Products in Spain and the Catalonia Region
Source: Toxins (Basel). 2022 Dec 29;15(1):24. doi: 10.3390/toxins15010024 (PMC9863481; doi:10.3390/toxins15010024)

Supplementary Materials: Occurrence and Dietary Exposure Assessment to Enniatin B through Consumption of Cereal-Based Products in Spain and the Catalonia Region

Jose A. Gallardo, Sonia Marín, Antonio J. Ramos, German Cano-Sancho and Vicente Sanchis

Table S1. Normalised consumption (g/kg bw/day) of cereal-based products for adult population from Catalonia Region and for all Spain. Mean levels and standard deviation (SD) were computed considering only consumers.

	Catalonia Region					Spain				
**Food**	**Age group**	**N total**	**% cons**	**Mean**	**SD**	**Age group**	**N total**	**% cons**	**Mean**	**SD**
White bread	18-40	864	61%	1.04	0.64	18-40	281	87%	1.17	0.84
	41-60	397	65%	1.23	0.59	41-60	209	89%	1.13	0.67
	>60	294	66%	1.26	0.58	>60	313	87%	1.07	0.70
Whole bread	18-40	864	29%	1.03	0.63	18-40	281	5%	0.70	0.55
	41-60	397	30%	1.10	0.65	41-60	209	12%	1.00	0.81
	>60	294	29%	1.09	0.65	>60	313	12%	0.71	0.66
Sliced bread	18-40	864	32%	0.39	0.27	18-40	281	34%	0.68	0.32
	41-60	397	22%	0.36	0.24	41-60	209	17%	0.66	0.33
	>60	294	22%	0.30	0.19	>60	313	18%	0.61	0.22
Bread rusks	18-40	864	21%	0.06	0.04	18-40	281	2%	0.24	0.21
	41-60	397	26%	0.06	0.04	41-60	209	3%	0.21	0.06
	>60	294	25%	0.05	0.04	>60	313	2%	0.24	0.06
Crackers	18-40	864	22%	0.01	0.01	18-40	281	5%	0.24	0.16
	41-60	397	18%	0.01	0.01	41-60	209	5%	0.21	0.16
	>60	294	15%	0.01	0.01	>60	313	3%	0.21	0.11
Cookies	18-40	864	20%	0.18	0.11	18-40	281	24%	0.50	0.34
	41-60	397	26%	0.16	0.09	41-60	209	20%	0.46	0.29
	>60	294	37%	0.14	0.10	>60	313	19%	0.36	0.18
Other cookies	18-40	864	38%	0.52	0.37	18-40	281	15%	0.75	0.68
	41-60	397	35%	0.45	0.28	41-60	209	16%	0.49	0.31

	>60	294	30%	0.39	0.26	>60	313	12%	0.46	0.31
Muffins	18-40	864	19%	0.46	0.32	18-40	281	10%	0.68	0.36
	41-60	397	22%	0.52	0.37	41-60	209	13%	0.64	0.40
	>60	294	28%	0.41	0.25	>60	313	8%	0.60	0.33
Cake	18-40	864	32%	0.47	0.23	18-40	281	7%	0.89	0.44
	41-60	397	27%	0.51	0.35	41-60	209	7%	1.19	0.66
	>60	294	26%	0.47	0.27	>60	313	8%	1.01	0.52
Breakfast cereals	18-40	864	34%	0.59	0.15	18-40	281	4%	0.48	0.26
	41-60	397	24%	0.53	0.13	41-60	209	2%	0.50	0.29
	>60	294	24%	0.50	0.13	>60	313	4%	0.49	0.21
Pasta	18-40	864	85%	0.54	0.20	18-40	281	44%	0.81	0.48
	41-60	397	75%	0.45	0.18	41-60	209	42%	0.64	0.41
	>60	294	84%	0.40	0.16	>60	313	37%	0.64	0.41

Table S2. Inventory of individual samples and composites prepared for the analysis for each food group.

	**Individual samples**	**Analytical composite samples**
White bread	68	4
Wholemeal bread	59	4
Sliced bread	22	3
Bread rusks	20	3
Crackers	21	3
Cookies	21	3
Other cookies	22	3
Muffins	20	3
Cakes	22	3
Breakfast cereals	20	3
Pasta	20	3
Baby food	32	32*
Total	347	63

* Baby food samples were analysed individually.

Figure S1. Contribution of cereal-based products to exposure of enniatin B to the Spanish population of 18-40 years old group (A), 41-60 years old group (B) and >60 years old group (C).

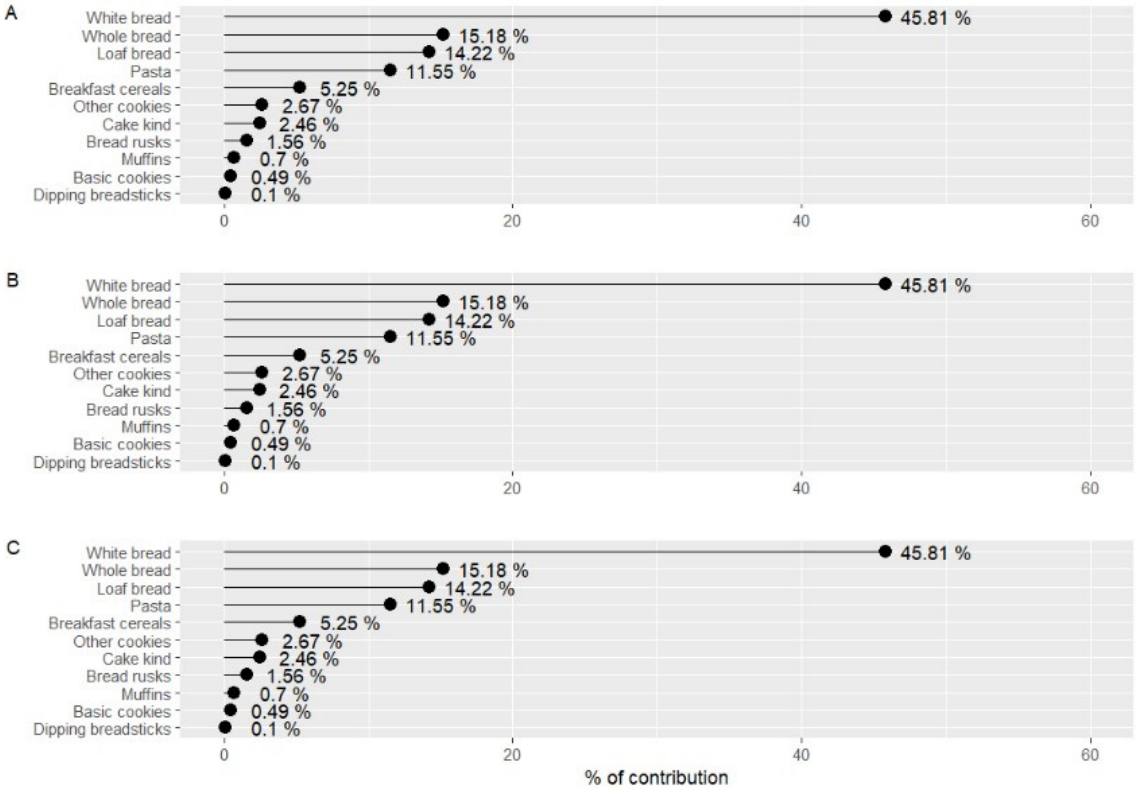

Figure S2. Contribution of cereal-based products to exposure of enniatin B to the 0-11 months old group (A), 12-35 months old group (B), 3-9 years old group (C) and 10-17 years old group (D).

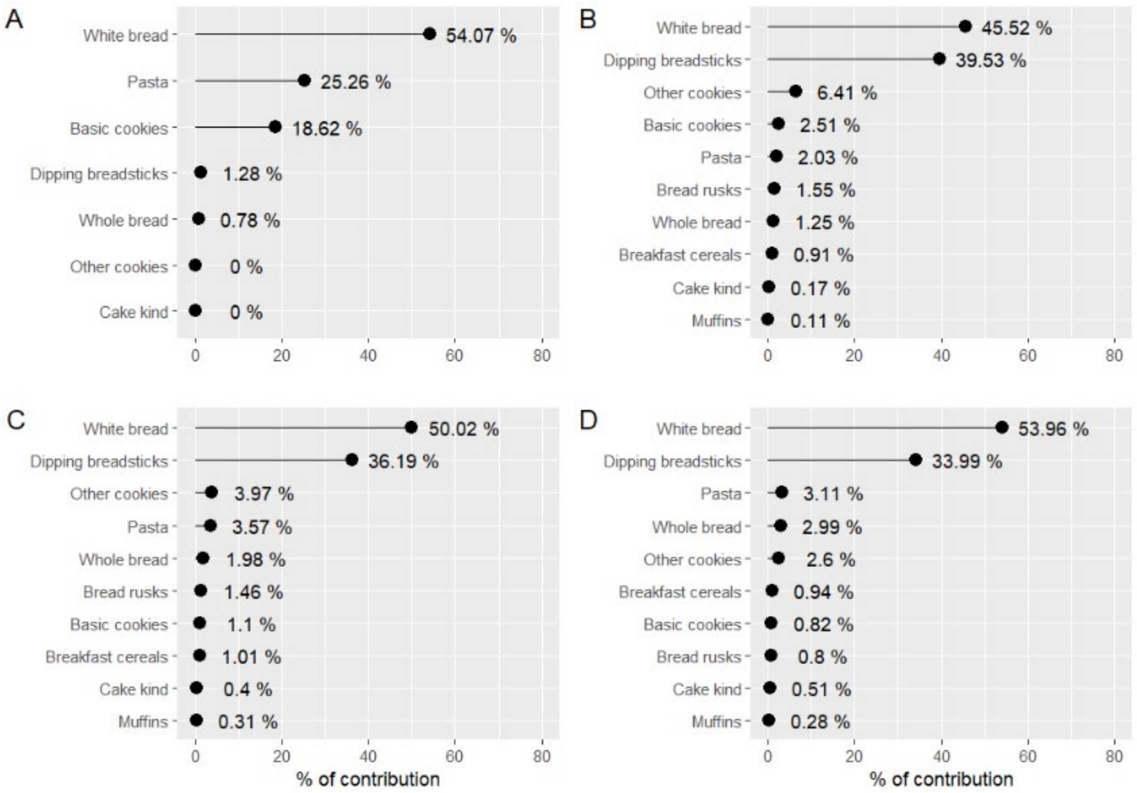

Supplement: Supplementary file 1 [file toxins-15-00024-s001.zip › toxins-2094057-supplementary.pdf]
